# Motivations, Facilitators, and Barriers of Donation-Based Interventions in HIV and Sexually Transmitted Infection Research: A Systematic Review

**DOI:** 10.1001/jamanetworkopen.2025.37382

**Published:** 2025-10-14

**Authors:** Dorian Ho, Ye Liu, Jamie Conklin, Thomas Fitzpatrick, Jiayu Wang, Suzanne Day, Takhona G. Hlatshwako, Rohit Ramaswamy, Ruby Congjiang Wang, Eneyi E. Kpokiri, Weiming Tang, Elvin Geng, Joseph D. Tucker

**Affiliations:** 1 Department of Health Policy and Management, Gillings School of Global Public Health, University of North Carolina at Chapel Hill, Chapel Hill; 2 University of North Carolina Project-China, Guangzhou, China; 3 Dermatology Hospital of South Medical University, Guangzhou, China; 4 Health Sciences Library, University of North Carolina at Chapel Hill, Chapel Hill; 5 School of Medicine, University of Washington, Seattle; 6 Division of Infectious Diseases, Department of Health Behavior, Gillings School of Global Public Health, University of North Carolina at Chapel Hill, Chapel Hill; 7 Department of Medicine, Division of Infectious Diseases, University of North Carolina at Chapel Hill, Chapel Hill; 8 James M. Anderson Center for Health Systems Excellence, Cincinnati Children’s Hospital Medical Center, Cincinnati, Ohio; 9 Clinical Research Department, Faculty of Infectious and Tropical Diseases, London School of Hygiene and Tropical Medicine, London, United Kingdom; 10 Division of Infectious Diseases, Washington University School of Medicine, St Louis, Missouri; 11 Institute of Global Health and Infectious Diseases, University of North Carolina at Chapel Hill, Chapel Hill

## Abstract

**Importance:**

Donation-based prosocial interventions involve someone receiving a free health service and then distributing or donating to support health services for others; examples within the HIV and sexually transmitted infection (STI) literature include secondary distribution of HIV self-tests, secondary syringe exchange, and pay it forward for STI testing. These interventions answer research and policy recommendations to incorporate prosocial behaviors into HIV/STI services.

**Objective:**

To describe motivations, facilitators, and barriers of donation-based interventions in HIV and STI research using data from qualitative studies.

**Evidence Review:**

In this systematic review, 5 databases (PubMed, CINAHL, Embase, PsycInfo, and Scopus) and references were searched up to January 23, 2024, for qualitative studies of donation-based interventions. Thematic synthesis was used to summarize findings, the Critical Appraisal Skills Programme Qualitative Studies Checklist was used to assess risk of bias among studies, and GRADE-CERQual (Confidence in the Evidence From Reviews of Qualitative Research) was used to assess confidence in review findings.

**Findings:**

Of 374 studies screened, 27 were included, which included 1543 participants, assessing secondary distribution of HIV self-tests (15 studies), secondary syringe exchange among people who inject drugs (10 studies), and pay it forward for STI testing (2 studies). Studies were from low-income (5 studies), middle-income (13 studies), and high-income (12 studies) countries. Givers who distributed health services were motivated by a selfless concern to benefit others (20 studies, moderate confidence) and by the cultivation of a prosocial identity (20 studies, moderate confidence). Social proximity between givers and recipients facilitated distribution (22 studies, moderate confidence), allowing for recipient-tailored strategies to introduce the service, strengthen peer relationships, and promote reciprocal giving. However, secondary syringe distribution could subject people who use drugs to legal harms and encourage them to provide unsupervised clinical care (7 studies, low confidence).

**Conclusions and Relevance:**

This systematic review identified motivations, facilitators, and barriers of donation-based interventions for HIV/STI services that could enhance implementation. Donation-based interventions may foster prosocial motivation and responsibility among socially marginalized populations to increase access to HIV/STI services.

## Key Points

**Question:**

What are the motivations, facilitators, and barriers of prosocial behavior in donation-based interventions in HIV and sexually transmitted infection research?

**Findings:**

This systematic review of 27 qualitative studies of donation-based interventions, which included 1543 participants, found that givers leveraged altruism, agency, and relationality with recipients to improve distribution and use of health services in their social networks. Distributing or donating services to others could foster a prosocial identity that increased givers’ concern and responsibility for others’ health needs.

**Meaning:**

Findings suggested that donation-based interventions could improve service uptake among marginalized populations using psychosocial assets already within those networks.

## Teaser

This systematic review uses data from qualitative studies to identify motivations, facilitators, and barriers of prosocial behavior for donation-based interventions in HIV and sexually transmitted infection research.

## Introduction

Collective action is required to effectively respond to pandemics. Following the COVID-19 pandemic, a Lancet Commission called for infectious disease interventions to foster prosocial attitudes.^1^ Prosociality manifests in voluntary acts that benefit others, such as informing, comforting, sharing, and helping.^2^ These behaviors can promote health formally in public health interventions and informally in social networks.^3^ To prevent HIV and other sexually transmitted infections (STIs), prosocial behaviors may be personal (eg, self-testing with altruistic intent to not infect others) or interpersonal (eg, organizing mutual aid for individuals living with HIV).^4,5^

Gift-giving is a universal prosocial behavior that builds and organizes social relationships through donation.^6,7,8^ Gift-giving fosters social ties through inclinations to give, receive, and reciprocate.^6,7,8^ Gift-giving features within a larger system of prosocial and social network interventions.^3,9^ However, gift-giving remains understudied in health promotion compared with prosocial interventions leveraging gratitude, charitable donations, and volunteerism.^3,10,11^

Social network interventions can incorporate gift-giving to improve the reach and uptake of HIV and STI services among socially marginalized populations. We define donation-based prosocial interventions, hereafter referred to as donation-based interventions, as someone receiving a free health service and then directly distributing or indirectly donating toward health services for others (eFigures 1 and 2 in Supplement 1). Examples of donation-based interventions in HIV and STI research include secondary distribution of HIV self-test (HIVST) kits, secondary syringe exchange among people who inject drugs (PWID) to prevent syringe reuse, and pay it forward to improve STI test uptake. Secondary distribution involves participants giving health services (HIVST kits, syringes) to persons in their social or sexual networks.^12,13^ Pay it forward involves a participant being given a free health service and offered the opportunity to donate money to support another participant’s health service.^14,15,16^

Donation-based interventions such as the secondary distribution of HIVST kits are effective in increasing test uptake and are supported by the World Health Organization and the Centers for Disease Control and Prevention.^3,12,17,18,19^ However, few studies have qualitatively explored the prosocial processes underlying donation-based interventions,^9,19^ nor have these insights been synthesized across intervention types. Additionally, most literature on incentivizing HIV/STI service uptake has studied financial incentives rather than social influences.^20,21,22^ Therefore, this systematic review and qualitative evidence synthesis aimed to describe the motivations, barriers, and facilitators of donation-based interventions in HIV and STI research.

## Methods

This systematic review was guided by the Preferred Reporting Items for Systematic Reviews and Meta-analyses (PRISMA) reporting guideline and the Cochrane Handbook.^23,24^ The review was prospectively registered in PROSPERO (CRD42024499448). Items are reported using the Enhancing the Transparency in the Reporting of Qualitative Health Research (ENTREQ) statement (eChecklist).^25^ Ethical approval was not needed for reviewing published literature.

Five databases (PubMed, CINAHL, Embase, PsycInfo, and Scopus) were searched from database inception to January 23, 2024. References of included studies and additional studies identified by the authors were hand searched. Searches were limited to peer-reviewed studies or gray literature published in English without date restrictions. The preplanned search strategy, developed with a research librarian (J.C.), included terms in the title and abstract describing donation-based interventions, such as secondary distribution, social network distribution, and pay it forward, and terms related to reciprocity (eAppendix in Supplement 1).

Two reviewers (D.H., Y.L.) independently screened titles and abstracts and then full texts to finalize articles for inclusion. Conflicts were resolved through consultation with a third reviewer (J.D.T.). The population of interest was participants in donation-based interventions for HIV/STI care. Formative, hypothetical, and preimplementation studies were excluded. Interventions had to fit the operational definition of a donation-based intervention in which someone received and used a free health service and then distributed or donated toward someone else’s health services. Additional criteria are provided in eFigure 2 in Supplement 1.

### Data Analysis

For data analysis, thematic synthesis was performed on the full text of included studies to inductively identify themes.^26^ Analysis followed the 3-step framework of Thomas and Harden.^26^ Two reviewers (D.H., Y.L.) independently used line-by-line coding through NVivo (release 1.7.2) to assign descriptive themes. New codes were created when existing codes were insufficient. Then, as a group, 3 reviewers (D.H., Y.L., J.D.T.) formed analytical themes interpreting the descriptive themes to address research questions. Themes were organized into motivations, facilitators, and barriers. Motivations referred to givers’ reasons for engaging in distribution, while facilitators and barriers referred to contextual factors that made introduction, receipt, and use of the distributed service easier or more difficult.

Two reviewers (D.H., Y.L.) independently assessed study quality using the Critical Appraisal Skills Programme Qualitative Studies Checklist.^27^ After finalizing analytical themes, 1 reviewer (D.H.) independently assessed confidence in selected review findings using GRADE-CERQual (Confidence in the Evidence From Reviews of Qualitative Research), which assesses methodological limitations, coherence, adequacy, and relevance.^28^ Results were checked by a second reviewer (J.D.T.).

## Results

### Study Characteristics

Our search identified 27 studies, which included 1543 participants, reporting qualitative evidence on 3 donation-based interventions (Figure). Interventions included secondary distribution of HIVST kits (15 studies),^29,30,31,32,33,34,35,36,37,38,39,40,41,42,43^ secondary syringe exchange among PWID (10 studies),^44,45,46,47,48,49,50,51,52,53^ and pay it forward for STI testing among men who have sex with men (MSM) (2 studies).^54,55^ Studies were from low-income (5 studies),^34,35,37,38,43^ middle-income (13 studies),^29,31,32,33,34,35,36,38,40,41,42,54,55^ and high-income (12 studies)^30,39,44,45,46,47,48,49,50,51,52,53^ countries (Table 1). Additional study characteristics are shown in Table 1, with more detail in eTable 1 in Supplement 1. Representative quotes are provided in eTable 2 in Supplement 1.

**Figure.**
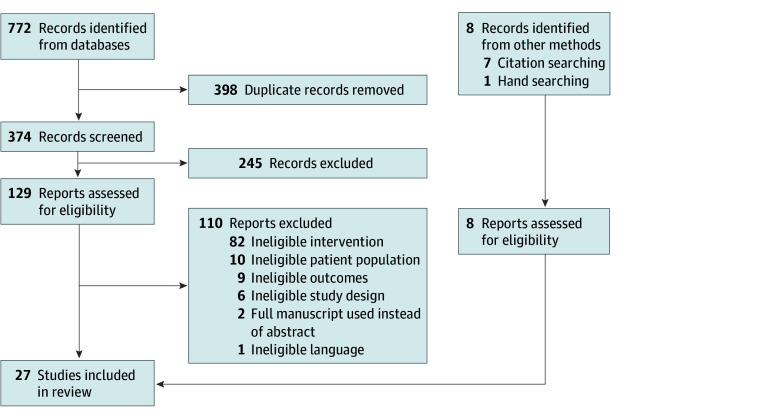
Preferred Reporting Items for Systematic Reviews and Meta-Analyses (PRISMA) Study Flow Diagram All reports sought for retrieval were retrieved.

**Table 1.** Included Study Demographics

Characteristic	Studies, No. (%)^a^
Total No.	27
Intervention	
Secondary distribution of HIV self-test kits	15 (56)
Secondary distribution of sterile syringes	10 (37)
Pay it forward for STI test uptake	2 (7)
Key population	
People who use drugs	11 (41)
Female sex workers	7 (26)
Heterosexual partners	6 (22)
Men who have sex with men	5 (19)
Pregnant women	2 (7)
Transgender individuals	1 (4)
Adolescent girls and young women	1 (4)
Country setting	
Low income	5 (19)
Middle income	13 (48)
High income	12 (44)
World Health Organization region	
African Region	11 (41)
Region of the Americas	8 (30)
Western Pacific Region	7 (26)
European Region	1 (4)

Abbreviation: STI, sexually transmitted infection.

^a^ Percentages may not sum to 100% because some studies included multiple populations or settings.

### Motivations for Distribution

#### Altruism and Morality

Of the 27 included studies, 20 observed altruism motivating givers to undertake donation-based interventions.^29,31,32,33,34,35,36,37,42,44,45,46,47,48,50,51,52,53,54,55^ These altruistic motivations focused on protecting the health of individuals in the same social and sexual networks as the giver. Givers were concerned about HIV transmission in their communities, motivating their decision to protect peers, partners, and others.^29,32,33,42,44,46,51^ Altruism could be explicit or implicit, such as wanting sexual partners to know their serostatus and preventing bloodborne virus transmission among PWID.^29,31,32,33,34,35,36,37,42,44,45,46,47,48,49,50,51,54^

Altruistic motivations often carried moral weight. Givers saw a moral obligation to help their recipients, either because of social roles or perceived needs. Female sex workers (FSW) and female partners felt it necessary that their sexual partners knew their own HIVST serostatus, prompting willingness to distribute HIVST kits.^29,32,33,34,35,36^ Many individuals who distributed syringes to other PWID felt it normatively unacceptable for syringes to be reused.^44,45,50,51^ Some obtained syringes for persons who were unable to do so due to housing insecurity, limited transportation, or chronic illness.^51,53^ In one context in which secondary syringe exchange was illegal, PWID defended their engagement in the practice as a necessary altruistic impulse to prevent infectious disease.^50^

#### Agency and Empowerment

Of the 27 included studies, 20 observed that donation-based interventions provided givers with agency in promoting informed health decisions and taking on prosocial roles.^29,30,31,32,33,34,35,36,38,40,46,47,48,49,50,51,52,53,54,55^ By distributing health services, givers felt empowered as responsible agents to promote healthier sexual and injection decisions. Secondary distribution of HIVST kits could enable givers to use their partner’s serostatus to make and justify safer sexual decisions. Serosorting techniques included condom negotiation, abstinence, or ending sexual relationships with individuals who refused to test or were serodiscordant.^29,30,31,35,36,40^ Negative HIV tests from both partners could inform condom use,^29,30,31,33,36,39,40^ although this was not universal.^31,34,36,39,40,41^ When conducting secondary syringe exchange, PWID not only collected and replaced used syringes, but also disseminated harm reduction information, taught safe injection practices, and dissuaded potential users from injecting.^46,47,48,49,50,51,53^

Serving as givers empowered key populations to challenge social stereotypes through prosocial roles. Secondary syringe exchange enabled PWID to proudly take on prosocial identities, countering stereotypes of PWID as irresponsible and fostering interpersonal accountability.^33,47,51,52^ Distribution was especially empowering for PWID with past medical training, fostering a continuity of identity between previous caring roles and caring for other PWID.^44,47^ For FSW, secondary distribution of HIVST kits enabled them to insist on condom usage and refuse sex without testing, restructuring gendered power dynamics to promote sexual autonomy.^34,35,36,40^ MSM felt that pay-it-forward participation enabled them to discreetly support each other and overcome the fragmentation of community.^54^

#### Transactional Motives

Of the 27 included studies, 8 mentioned transactional motives for engaging in donation-based interventions.^34,35,41,44,51,52,53,55^ Distribution was sometimes leveraged for givers’ financial or material benefit. FSW could use HIVST kit distribution to negotiate higher prices for services.^34,35^ PWID infrequently reported exchanging syringes for drugs or money.^44,51,52,53^ Research incentives for HIVST kit distribution could foster financial rather than prosocial reasons for distribution.^41^

### Facilitators of Distribution

#### Social Norms of Sharing

Of the 27 included studies, 8 observed that donation-based interventions were facilitated by leveraging social norms of resource sharing among similarly marginalized communities.^35,44,47,48,51,52,54,55^ PWID often mentioned sharing resources beyond clean syringes, such as providing food, shelter, money, and help with errands to persons in the same building or social circles.^44,47,51,52^ However, this ethic of sharing sometimes facilitated the sharing of used syringes.^48^ Secondary syringe exchange could replace these risk behaviors while drawing on the same social norm of sharing limited resources.^48^ Apart from PWID, MSM and FSW informally distributed HIVST kits among persons with similar social identities, seeing themselves at similar risk of HIV.^35^ MSM in pay-it-forward interventions perceived an obligation to donate to other MSM, acknowledging a common identity and knowing that other MSM subsidized their test.^54,55^

#### Social Proximity and Reciprocity

Of the 27 included studies, 22 observed how social proximity between the giver and recipient facilitated service uptake.^29,30,31,32,33,34,35,36,37,38,39,40,41,42,43,44,45,46,48,50,51,52^ Conversely, distribution without an existing social relationship was uncommon.^34,42,45,51^ Social proximity provided opportunities to introduce the service, strengthen relationships, and engage in reciprocal giving.

For HIVST kit distribution, givers’ closeness to their recipients enabled them to be innovative and intentional in how they introduced the gift. Givers knew recipients’ schedules and preferences, thus leveraging opportune times, visible locations, and persuasion to introduce the test.^29,30,32,35,36,37,39,41,43^ Givers often tested together or shared their HIV status to build trust.^29,35,36,41^ However, they sometimes concealed the purpose of the HIVST kit, especially for individuals fearful of disclosing their HIV status.^35,37,43^

Secondary distribution of HIVST kits strengthened relationships among long-term partners, but reactive results could dissolve short-term relationships. For couples, use of HIVST kits could convey care and commitment for the partner’s health.^29,32,33,37,39,40^ Among serodiscordant couples, knowledge of each partner’s serostatus could facilitate acceptance, with male partners encouraging female partners with HIV to seek care.^29,37^ However, for casual partners, clients, and some couples, reactive tests or refusal to test could result in abstinence, distancing, or abandonment.^30,31,32,36,40^

Distribution could also result in reciprocity from the recipient. Reciprocal action was frequently directed toward the giver, including gratitude and gifts for HIVST kits or drugs and favors for new syringes.^32,40,51,53^ Syringe recipients could also provide givers with new syringes in future interactions, indicating role fluidity.^50^ In 2 studies, reciprocal action could be paid forward within social networks, such as tertiary distribution from the original recipient to new recipients.^35,50^ For pay-it-forward interventions, this reciprocity formed the basis of the intervention itself.^54,55^

The absence of trust and commitment could render distribution unsuccessful. HIVST kit distribution was generally not attempted in relationships with interpersonal violence.^33^ Having female partners offer HIVST kits to males with multiple partners led to mistrust and refusal arising from suspected infidelity from the female partner.^33^ For secondary syringe exchange, PWID were hesitant to accept syringes from PWID outside their social circles.^45,51^

#### Simple Instructions and Knowledge

Simple health service instructions and givers’ knowledge facilitated distribution in 15 of the 27 included studies.^29,31,32,33,34,35,38,39,40,41,43,44,46,51,52^ For HIVST kits, givers were almost always able to explain self-testing procedures, refer to instructions, and provide assistance to recipients.^29,31,32,33,34,35,38,39,40,41,43^ Givers were knowledgeable about procedures for administering HIVST kits, such as collecting samples and waiting for results.^29,31,32,34,35,40,43^ If unsure, they could readily access instructions received from the original point of care, such as a clinic or community health worker.^34,35,38^ Givers sometimes discussed sexual risk reduction during distribution.^35,36^

For secondary syringe exchange, distribution required little additional education, as givers were generally knowledgeable of safe injection practices.^44,46,51,52^ Alongside offering clean syringes, some givers distributed other harm reduction supplies (eg, cookers, cottons, condoms, alcohol wipes, and biohazard containers), collected used syringes, and prevented reuse by breaking needles.^48,50,51,52^

### Barriers to Distribution

#### Burden of Responsibility

Distributing new syringes through secondary syringe exchange could lead to unofficial responsibilities and legal harms in 7 of the 10 studies on this intervention.^46,47,48,50,51,52,53^ As documented in 2 studies, secondary syringe exchange could lead to informal doctoring, in which givers would be called on to manage care needs beyond their scope of responsibility or expertise.^47,51^ These unofficial roles often began by providing injection assistance to PWID whose veins were difficult to access.^47,51^ These roles could progress to monitoring drug tolerance, managing overdoses, distributing antibiotics, and providing wound and abscess care.^47,51^ Though givers were willing to take on these responsibilities, they faced moral distress when the medical care needed exceeded their training and resources.^47^ These situations were compounded by the reluctance of recipient PWID to involve medical authorities or law enforcement.^47^

In 7 studies, givers taking on the officially sanctioned role of distributing new syringes could be confronted by law enforcement.^46,47,48,50,51,52,53^ In settings with prohibitionist legal attitudes toward drug use, syringe possession could attract attention from police, neighbors, and child protective services.^46,47,48,50,51,52,53^ Secondary syringe exchange could thus shift these risks from syringe exchange programs onto givers, even in settings where secondary syringe exchange was legally permissible.^46^

Conversely, givers did not appear overly burdened in HIVST kit and pay-it-forward interventions. They capably administered tests, managed recipient emotions, and handled reactive results.^30,31,39^ However, 1 study discussed secondary distribution as burdensome for pregnant women, especially when handling negative partner reactions.^43^

#### Social Harms

Of the 15 studies on the secondary distribution of HIVST kits, 5 examined associated social harms.^32,35,36,38,39^ Physical and sexual abuse were observed in 5 studies, although not always because of HIVST kit distribution.^32,35,36,38,39^ Relational dissolution was threatened in 2 studies and occurred in another 2 studies.^32,35,36,37^ However, harms from HIVST kit distribution were generally rare.

#### Confidence in Findings

Seventeen studies^29,30,31,32,33,35,36,37,39,40,41,42,43,47,48,50,52^ were rated as having minor methodological limitations (Table 2). The most common limitations were lack of reflexivity and limited mention of ethical considerations. Four major findings were assessed using GRADE-CERQual for confidence in findings (Table 3). There was moderate confidence in review findings on motivations and facilitators, although the barrier identified for secondary syringe exchange was rated low confidence.

**Table 2.** CASP Qualitative Studies Checklist

Source	Clear aims?	Qualitative methodology appropriate?	Research design appropriate?	Recruitment appropriate?	Data collection appropriate?	Reflexivity considered?	Ethical issues considered?	Data analysis rigorous?	Clear findings?	Practice, policy implications?	Research implications?	Transferability discussed?	Rating
Agot et al,^29^ 2020	Yes	Yes	Yes	Yes	Yes	Unclear	Yes	Yes	Yes	Yes	No	Yes	Minor
Balán et al,^30^ 2020	Yes	Yes	Yes	Yes	Yes	Unclear	Yes	Yes	Yes	Yes	Yes	Yes	Minor
Boisvert Moreau et al,^31^ 2022	Yes	Yes	Yes	Yes	Yes	Unclear	Yes	Yes	Yes	Yes	No	Yes	Minor
Brothers,^44^ 2016	Yes	Yes	Yes	Yes	Yes	Yes	Yes	Yes	Yes	Yes	Yes	Yes	None or very minor
Bryant et al,^45^ 2009	Yes	Yes	Unclear	No	Yes	Unclear	Unclear	Unclear	Yes	Yes	Yes	Yes	Major
Bryant et al,^46^ 2019	Yes	Yes	Yes	Yes	Yes	Unclear	Unclear	Unclear	Yes	Yes	No	No	Moderate
Bwalya et al,^32^ 2020	Yes	Yes	Yes	Yes	Yes	Unclear	Yes	Yes	Yes	Yes	Yes	Yes	Minor
Byrne et al,^54^ 2024	Yes	Yes	Yes	Yes	Yes	Yes	Yes	Yes	Yes	Yes	Yes	Yes	None or very minor
Dechman^47^ 2015	Yes	Yes	Yes	Yes	Yes	Yes	Yes	Yes	Yes	Yes	No	No	Minor
Grund et al,^48^ 1992	Yes	Yes	Yes	Yes	Yes	Yes	Unclear	Yes	Yes	Yes	Yes	No	Minor
Holmes et al,^33^ 2020	Yes	Yes	Yes	Yes	Yes	Unclear	Yes	Yes	Yes	Yes	Yes	Yes	Minor
Ky-Zerbo et al,^35^ 2023	Yes	Yes	Yes	No	Yes	Yes	Yes	Yes	Yes	Yes	Yes	Yes	Minor
Ky-Zerbo et al,^34^ 2022	Yes	Yes	Yes	Yes	Yes	Yes	Yes	Yes	Yes	Yes	No	Yes	None or very minor
Li et al,^55^ 2020	Yes	Yes	Yes	No	Yes	Unclear	Yes	Unclear	Yes	Yes	Yes	Yes	Moderate
Maman et al,^36^ 2017	Yes	Yes	Yes	Yes	Yes	Unclear	Yes	Yes	Yes	Yes	Yes	Yes	Minor
Matovu et al,^37^ 2018	Yes	Yes	Yes	Yes	Yes	No	Unclear	Yes	Yes	Yes	Yes	Yes	Minor
Murphy et al,^49^ 2004	Yes	Yes	Yes	Yes	Unclear	Unclear	Unclear	Unclear	Yes	Yes	No	No	Major
Napierala et al,^38^ 2019	Yes	Yes	Yes	Yes	Unclear	Unclear	Unclear	No	Yes	Yes	Yes	Yes	Moderate
Newland et al,^50^2016	Yes	Yes	Yes	Yes	Yes	Unclear	Yes	Yes	Yes	Yes	Yes	Yes	Minor
Rael et al,^39^ 2020	Yes	Yes	Yes	No	Yes	Unclear	Unclear	Yes	Yes	Yes	Yes	Yes	Minor
Ruderman et al,^40^ 2022	Yes	Yes	Yes	Yes	Yes	Unclear	Yes	Yes	Yes	Yes	Yes	Yes	Minor
Sha et al,^41^ 2023	Yes	Yes	Yes	Yes	Yes	Unclear	Yes	Yes	Yes	Yes	Yes	Yes	Minor
Snead et al,^51^ 2003	Yes	Yes	Yes	Yes	Yes	Yes	Yes	Yes	Yes	Yes	Yes	Yes	None
Strike et al,^52^ 2005	Yes	Yes	Yes	Yes	Yes	Unclear	Unclear	Yes	Yes	Yes	Yes	No	Minor
Voytek et al,^53^ 2003	Yes	Yes	Yes	Unclear	Unclear	Unclear	Unclear	Unclear	Yes	Yes	No	No	Major
Wang et al,^42^ 2024	Yes	Yes	Yes	Yes	Yes	Unclear	Yes	Yes	Yes	Yes	Yes	No	Minor
Ware et al,^43^ 2023	Yes	Yes	Yes	Yes	Yes	Unclear	Yes	Yes	Yes	Yes	Yes	Yes	Minor

Abbreviation: CASP, Critical Appraisal Skills Programme.

**Table 3.** GRADE-CERQual Assessment

Key finding	Assessment categories				Rating and comments
	Methodological limitations	Coherence	Adequacy	Relevance	
Altruism and moral obligation motivated givers to distribute health services.^29,31,32,33,34,35,36,37,42,44,45,46,47,48,50,51,52,53,54,55^	Minor concerns (12 studies with minor limitations, 2 studies with moderate limitations, 2 studies with major limitations, with most studies lacking reflexivity)	Moderate concerns (9 studies indicated egoistic motivations for distribution, such as personal protection for HIVST kit, financial gain for syringes)	Minor concerns (20 studies, most with moderately rich data)	No or very minor concerns (range of study settings and populations, 1 study that included some participants who had not distributed tests)	Moderate: moderate concerns about adequacy given competing motivations, minor concerns about methodological limitations, adequacy
Givers were empowered as responsible agents who could make more informed decisions and challenge disempowering stereotypes.^29,30,31,32,33,34,35,36,38,40,46,47,48,49,50,51,52,53,54,55^	Minor concerns (12 studies with minor limitations, 3 studies with moderate limitations, 2 studies with major limitations, with most studies lacking reflexivity)	Moderate concerns (10 HIVST kit studies indicated unprotected sex after both partners tested HIV-negative)	Minor concerns (20 studies, most with moderately rich data)	No or very minor concerns (range of study settings and populations)	Moderate: moderate concerns about adequacy given willingness to eschew preventive behaviors, minor concerns about methodological limitations, adequacy
Social proximity and trust between giver and recipient facilitated distribution and reciprocity.^29,30,31,32,33,34,35,36,37,38,39,40,41,42,43,44,45,46,48,50,51,52^	Minor concerns (16 studies with minor limitations, 2 studies with moderate limitations, 1 study with major limitations, with most studies lacking reflexivity)	Minor concerns (3 studies indicated trust precluding the need for HIVST kits)	Moderate concerns (22 studies, most with thin data)	No or very minor concerns (range of study settings and populations)	Moderate: moderate concerns about adequacy, given thinness and heterogeneous description of relational factors across studies, minor concerns about methodological limitations, adequacy
Secondary syringe exchange could put a high burden of risk and responsibility on givers.^45,46,47,48,50,51,52,53^	Minor concerns (4 studies with minor limitations, 1 study with moderate limitations, 1 study with major limitations, with most studies lacking reflexivity)	No or very minor concerns	Moderate concerns (7 studies, all but Bryant and Hopwood^45^ and Dechman^47^ with thin data)	Moderate concerns (all studies came from high-income settings with established syringe exchange programs)	Low: moderate concerns about adequacy, given smaller pool of studies, moderate concerns about relevance given bias from high-income settings, minor concerns about methodological limitations

Abbreviations: CERQual, Confidence in the Evidence From Reviews of Qualitative Research; HIVST, HIV self-test.

## Discussion

This systematic review synthesized qualitative evidence on the prosocial processes underlying donation-based interventions in HIV/STI care. Donation-based interventions were motivated by moral emotions and psychosocial assets, such as altruism, agency, and social norms. These interventions were facilitated by givers’ embeddedness within social networks as well as by the simplicity of distributing health services and peer intuition on service use. However, secondary syringe exchange may have increased responsibility and induced moral and legal harms for givers. This systematic review extends existing literature by elucidating the behavioral context of donation-based interventions and describing barriers and facilitators. Our findings suggest that donation-based interventions leverage social networks and norms to fill gaps in HIV/STI service uptake among marginalized populations.

Givers’ motivations for distribution were found to be altruistic, moral, and community-oriented. Givers understood the health impacts of bloodborne illnesses and valued the health of peers in their social networks, echoing literature on collectivist motivations for HIV testing and avoiding syringe reuse.^56,57,58^ Recognizing and fostering altruistic intent appeared especially relevant for retaining givers and sustaining secondary distribution, as seen in literature on sustaining peer education programs.^59,60,61^ For secondary syringe exchange, altruistic motivations intersected with cultural norms of sharing limited communal resources among PWID.^62,63^ Secondary syringe exchange could reshape attitudes toward syringe reuse while applying the same moral economies of sharing.

Distribution also empowered givers by enabling them to take on prosocial roles. This finding is consistent with themes of agency and empowerment reported among peer educators and community health workers in other HIV/STI interventions.^64,65,66,67^ Empowerment from secondary distribution was frequently described among FSW and PWID. Because these groups practiced distribution most frequently, repeated giving behavior may foster prosocial identities and challenge disempowering stereotypes about these populations.^59^ Additionally, givers’ empowerment was not at recipients’ expense. Empowerment of peer educators has sometimes been criticized for disempowering clients because peer educators may create social distance with clients and view them lower on hierarchies of worth.^68^ Given the transient and informal nature of secondary distribution, the nature of empowerment with donation-based interventions appears mechanistically different. The embedded positions of givers within social networks suggests that donation-based networks may be more relationally aware and nonhierarchical. Future research should compare potential motivational differences between informal distribution and peer education.

We found secondary syringe exchange posed several risks for givers. Most importantly, the lack of institutional oversight may have put too much responsibility on givers. Previous studies have found PWID peer workers are pressured to go beyond professional roles and experience burnout when support and supervision are lacking.^69,70,71^ These issues indicate the tension between the informality needed to access unreached populations and practical limits on care outside formal health settings. Beyond additional training, givers require relationships with staff at syringe exchange programs and bridge clinics to promote recipient referral. Secondary syringe exchange also conflicts with prohibitive legal attitudes that stigmatize or criminalize possession of drug paraphernalia, imbuing distribution with legal risk.^72^ Other studies have found that secondary syringe exchange occurs alongside reuse of syringes,^73,74^ indicating that cultural norms of sharing needles are more entrenched than public health logics for preventing disease. Further qualitative investigation is warranted.

These findings have practice and policy implications. Donation-based interventions align with trends toward task-shifting and service integration in HIV/STI service delivery. These donation-based interventions extend task-shifting beyond peer education, with the role of a giver requiring less formal training and intentionally leveraging personal social networks. In line with service integration, HIV and STI self-tests, new syringes, and condoms could be bundled for distribution. Given the effectiveness of HIVST kit secondary distribution in trials and meta-analyses,^12,19^ these health services should be subsidized and readily available in clinical and community settings. However, the mixed evidence on using secondary syringe exchange to reduce needle-sharing behaviors requires resolution before adoption by exchanges.^72,73,74^

### Limitations

Our study has several limitations. First, generalizability for some findings is limited. For instance, all studies on secondary syringe exchange came from high-income countries. That said, evidence on HIVST kit secondary distribution covered a range of key populations and geographic settings. Second, evidence on pay it forward was underrepresented compared with other interventions, but many themes, including altruistic motivations for donation, agency from participation, and normative influences, agreed with other findings. In addition, studies tended to lack reflexivity, indicating biases in primary interpretation, although these limitations were considered when assessing confidence in review findings.

## Conclusions

In this systematic review, donation-based interventions drew on altruism, agency, and relationality between participants to improve HIV/STI service uptake. These interventions may foster prosocial motivation and responsibility among socially marginalized populations to increase access to HIV/STI services.

## Notes

Supplement 1. **eFigure 1.** Schematic of donation-based interventions **eFigure 2.** Typology of donation-based interventions **eAppendix.** Search strategy report **eTable 1.** Study demographics **eTable 2.** Representative quotes for themes

Supplement 2.
Data Sharing Statement
