# Supplementary material for: Motivations, Facilitators, and Barriers of Donation-Based Interventions in HIV and Sexually Transmitted Infection Research: A Systematic Review
Source: JAMA Netw Open. 2025 Oct 14;8(10):e2537382. doi: 10.1001/jamanetworkopen.2025.37382 (PMC12522005; doi:10.1001/jamanetworkopen.2025.37382)
Supplement: Supplement 1. — eFigure 1. Schematic of donation-based interventions eFigure 2. Typology of donation-based interventions eAppendix. Search strategy report eTable 1. Study demographics eTable 2. Representative quotes for themes [file jamanetwopen-e2537382-s001.pdf]

Supplementary Online Content

Ho D, Liu Y, Conklin J, et al. Motivations, facilitators, and barriers of donation-based interventions in HIV and sexually transmitted infection research: a systematic review. *JAMA Netw Open*. 2025;8(10):e2537382. doi:10.1001/jamanetworkopen.2025.37382

eFigure 1. Schematic of donation-based interventions
eFigure 2. Typology of donation-based interventions
eAppendix. Search strategy report
eTable 1. Study demographics
eTable 2. Representative quotes for themes

This supplementary material has been provided by the authors to give readers additional information about their work.

© 2025 Ho D et al. *JAMA Network Open*.

eFigure 1. Schematic of donation-based interventions

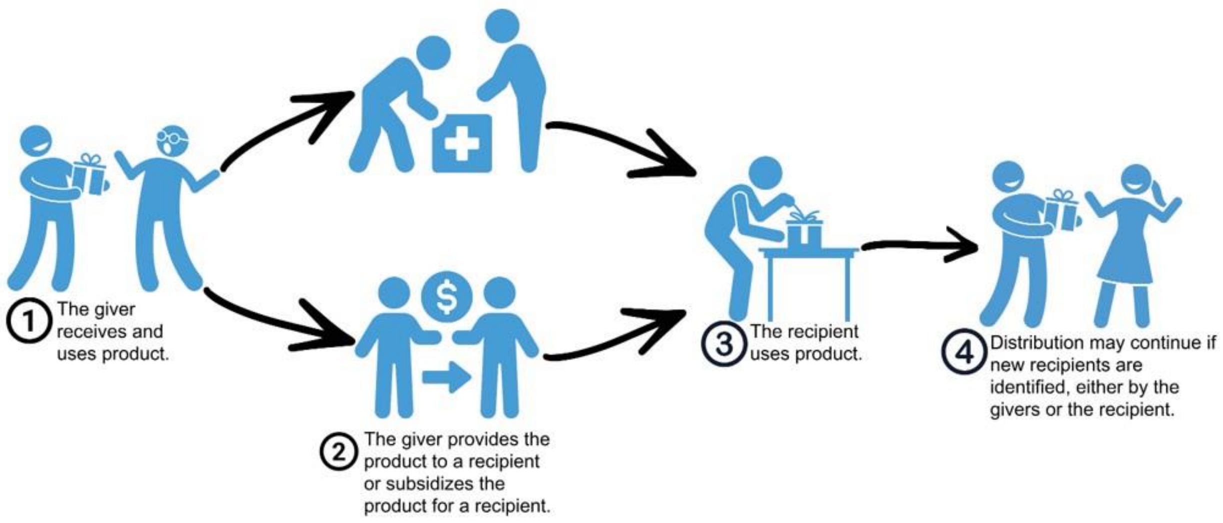

The giver refers to the participant who first receives the health service from a clinical or community setting, while the recipient refers to the social network member who receives a gifted health service from the giver.

© 2025 Ho D et al. *JAMA Network Open*.

eFigure 2. Typology of donation-based interventions

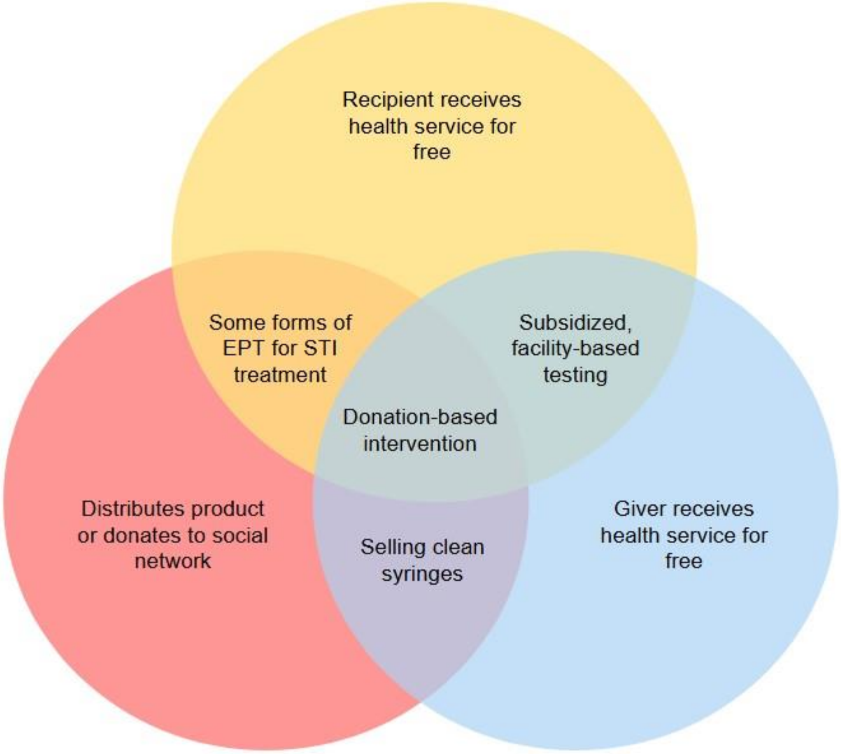

EPT = expedited partner therapy, STI = sexually transmitted infection. For this review, peer condom distribution was excluded because of substantial evidence supporting the approach.[75] Expedited partner therapy for STI treatment was excluded because of inconsistency in whether givers and recipients pay for the service,[76] and evidence syntheses already exist.[77,78] Interventions distributing vouchers or coupons rather than health services were excluded because they do not independently constitute a health service.

eAppendix. Search Strategy Report

Date: 1/23/2024

Total # of References: 772
# of Duplicates Removed: 390
Total # of References to Screen: 382

Database: PubMed

Set #		Results
1	("gift receipt"[TITLE/ABSTRACT:~0] OR "receiving gifts" [TITLE/ABSTRACT] OR "gratitude" [TITLE/ABSTRACT] OR "gratefulness" [TITLE/ABSTRACT] OR "thankfulness" [TITLE/ABSTRACT] OR "indebtedness" [TITLE/ABSTRACT])	
2	("gift-giving" [TITLE/ABSTRACT] OR "mutual aid" [TITLE/ABSTRACT] OR "donation" [TITLE/ABSTRACT] OR "donations" [TITLE/ABSTRACT] OR "volunteerism" [TITLE/ABSTRACT] OR "volunteering" [TITLE/ABSTRACT] OR "prosocial" [TITLE/ABSTRACT] OR "prosociality" [TITLE/ABSTRACT] OR "generosity" [TITLE/ABSTRACT] OR "altruism" [TITLE/ABSTRACT] OR "warm glow" [TITLE/ABSTRACT])	
3	#1 AND #2	
4	("pay it forward" [TITLE/ABSTRACT] OR pay-it-forward [TITLE/ABSTRACT] or "paying it forward" [TITLE/ABSTRACT] OR paying-it-forward [TITLE/ABSTRACT] OR "upstream reciprocity" [TITLE/ABSTRACT] OR "peer donation" [TITLE/ABSTRACT] OR "peer donations" [TITLE/ABSTRACT:~0] OR "peer distribution" [TITLE/ABSTRACT] OR "peer distributions" [TITLE/ABSTRACT:~0] OR "extended distribution" [TITLE/ABSTRACT] OR "extended distributions" [TITLE/ABSTRACT] OR "secondary distribution" [TITLE/ABSTRACT] OR "secondary distributions" [TITLE/ABSTRACT:~0] OR "secondary exchange" [TITLE/ABSTRACT] OR "secondary exchanges" [TITLE/ABSTRACT:~0] OR "kidney paired donation"[TITLE/ABSTRACT] OR "kidney paired donations"[TITLE/ABSTRACT] OR "paired kidney donation"[TITLE/ABSTRACT] OR "paired kidney donations"[TITLE/ABSTRACT] OR "paired kidney exchang*"[TITLE/ABSTRACT] OR "kidney paired exchang*"[TITLE/ABSTRACT])	
5	#3 OR #4	902
6	((("semi-structured"[TITLE/ABSTRACT] OR semistructured[TITLE/ABSTRACT] OR unstructured[TITLE/ABSTRACT] OR informal[TITLE/ABSTRACT] OR "in-depth"[TITLE/ABSTRACT] OR indepth[TITLE/ABSTRACT] OR "face-to-face"[TITLE/ABSTRACT] OR structured[TITLE/ABSTRACT] OR guide[TITLE/ABSTRACT] OR guides[TITLE/ABSTRACT]) AND (interview*[TITLE/ABSTRACT] OR discussion*[TITLE/ABSTRACT] OR questionnaire*[TITLE/ABSTRACT])) OR "focus group"[TITLE/ABSTRACT] OR "focus groups"[TW] OR qualitative[TITLE/ABSTRACT] OR "mixed methods"[TITLE/ABSTRACT] OR ethnograph*[TITLE/ABSTRACT] OR "key informant"[TITLE/ABSTRACT] OR "participant observation"[Title/Abstract] OR "participant observations"[Title/Abstract] OR anthropology[tw] OR anthropological[Title/Abstract] OR narrative[Title/Abstract] OR "grounded theory"[tw] OR "interviews as topic"[Mesh] OR narration[Mesh] OR "qualitative research"[Mesh] OR "personal narratives as topic"[Mesh] OR "anecdotes as topic"[Mesh])	
7	#5 AND #6	125
8	#7 AND English[lang]	123

Database: Embase (Elsevier)

Set #		Results
1	(‘gift receipt’:ti,ab,kw OR ‘receiving gifts’:ti,ab,kw OR ‘gratitude’:ti,ab,kw OR ‘gratefulness’:ti,ab,kw OR ‘thankfulness’:ti,ab,kw OR ‘indebtedness’:ti,ab,kw)	
2	(‘gift-giving’:ti,ab,kw OR ‘mutual aid’:ti,ab,kw OR ‘donation’:ti,ab,kw OR ‘donations’:ti,ab,kw OR ‘volunteerism’:ti,ab,kw OR ‘volunteering’:ti,ab,kw OR ‘prosocial’:ti,ab,kw OR ‘prosociality’:ti,ab,kw OR ‘generosity’:ti,ab,kw OR ‘altruism’:ti,ab,kw OR ‘warm glow’:ti,ab,kw)	
3	#1 AND #2	
4	(‘pay it forward’:ti,ab,kw OR pay-it-forward:ti,ab,kw OR ‘paying it forward’:ti,ab,kw OR paying-it-forward:ti,ab,kw OR ‘upstream reciprocity’:ti,ab,kw OR ‘peer donation’:ti,ab,kw OR ‘peer donations’:ti,ab,kw OR ‘peer distribution’:ti,ab,kw OR ‘peer distributions’:ti,ab,kw OR ‘extended distribution’:ti,ab,kw OR ‘extended distributions’:ti,ab,kw OR ‘secondary distribution’:ti,ab,kw OR ‘secondary distributions’:ti,ab,kw OR ‘secondary exchange’:ti,ab,kw OR ‘secondary exchanges’:ti,ab,kw OR ‘kidney paired donation’:ti,ab,kw OR ‘kidney paired donations’:ti,ab,kw OR ‘paired kidney donation’:ti,ab,kw OR ‘paired kidney donations’:ti,ab,kw OR ‘paired kidney exchang*’:ti,ab,kw OR ‘kidney paired exchang*’:ti,ab,kw)	
5	#3 OR #4	1424
6	(((‘semi-structured’:ti,ab,kw OR semistructured:ti,ab,kw OR unstructured:ti,ab,kw OR informal:ti,ab,kw OR ‘in-depth’:ti,ab,kw OR indepth:ti,ab,kw OR ‘face-to-face’:ti,ab,kw OR structured:ti,ab,kw OR guide:ti,ab,kw OR guides:ti,ab,kw) AND (interview*:ti,ab,kw OR discussion*:ti,ab,kw OR questionnaire*:ti,ab,kw)) OR ‘focus group’:ti,ab,kw OR ‘focus groups’:ti,ab,kw OR qualitative:ti,ab,kw OR ‘mixed methods’:ti,ab,kw OR ethnograph*:ti,ab,kw OR ‘key informant’:ti,ab,kw OR ‘participant observation’:ti,ab,kw OR ‘participant observations’:ti,ab,kw OR anthropology:ti,ab,kw OR anthropological:ti,ab,kw OR narrative:ti,ab,kw OR ‘grounded theory’:ti,ab,kw OR 'interview'/exp OR 'verbal communication'/exp OR 'qualitative research'/exp OR 'literature'/exp)	
7	#5 AND #6	221
8	#7 AND [english]/lim	218

Database: CINAHL Plus with Full Text (EBSCO*host*)

Set #		Results
1	TI (“gift receipt” OR “receiving gifts” OR “gratitude” OR “gratefulness” OR “thankfulness” OR “indebtedness”) OR AB (“gift receipt” OR “receiving gifts” OR “gratitude” OR “gratefulness” OR “thankfulness” OR “indebtedness”)	
2	TI (“gift-giving” OR “mutual aid” OR “donation” OR “donations” OR “volunteerism” OR “volunteering” OR “prosocial” OR “prosociality” OR “generosity” OR “altruism” OR “warm glow”) OR AB (“gift-giving” OR “mutual aid” OR “donation” OR “donations” OR “volunteerism” OR “volunteering” OR “prosocial” OR “prosociality” OR “generosity” OR “altruism” OR “warm glow”)	
3	#1 AND #2	
4	TI (“pay it forward” OR pay-it-forward or “paying it forward” OR paying-it-forward OR “upstream reciprocity” OR “peer donation” OR “peer donations” OR “peer distribution” OR “peer distributions” OR “extended distribution” OR “extended distributions” OR “secondary distribution” OR “secondary distributions” OR “secondary exchange” OR “secondary exchanges” OR “kidney paired donation” OR “kidney paired donations” OR “paired kidney donation” OR “paired kidney	

© 2025 Ho D et al. *JAMA Network Open*.

	donations” OR “paired kidney exchang*” OR “kidney paired exchang*”) OR AB (“pay it forward” OR pay-it-forward or “paying it forward” OR paying-it-forward OR “upstream reciprocity” OR “peer donation” OR “peer donations” OR “peer distribution” OR “peer distributions” OR “extended distribution” OR “extended distributions” OR “secondary distribution” OR “secondary distributions” OR “secondary exchange” OR “secondary exchanges” OR “kidney paired donation” OR “kidney paired donations” OR “paired kidney donation” OR “paired kidney donations” OR “paired kidney exchang*” OR “kidney paired exchang*”)	
5	#3 OR #4	451
6	(((TI (“semi-structured” OR semistructured OR unstructured OR informal OR “in-depth” OR indepth OR “face-to-face” OR structured OR guide OR guides) OR AB (“semi-structured” OR semistructured OR unstructured OR informal OR “in-depth” OR indepth OR “face-to-face” OR structured OR guide OR guides)) AND (TI (interview* OR discussion* OR questionnaire*) OR AB (interview* OR discussion* OR questionnaire*))) OR TI (“focus group” OR “focus groups” OR qualitative OR “mixed methods” OR ethnograph* OR “key informant” OR “participant observation” OR “participant observations” OR anthropology OR anthropological OR narrative OR “grounded theory”) OR AB (“focus group” OR “focus groups” OR qualitative OR “mixed methods” OR ethnograph* OR “key informant” OR “participant observation” OR “participant observations” OR anthropology OR anthropological OR narrative OR “grounded theory”) OR MH "Interviews+" OR MH "Narratives+" OR MH "Focus Groups" OR MH "Qualitative Studies+" OR MH "Storytelling")	
7	#5 AND #6	82
8	#7 AND English	81

Database: APA PsycInfo (EBSCO*host*)

Set #		Results
1	TI (“gift receipt” OR “receiving gifts” OR “gratitude” OR “gratefulness” OR “thankfulness” OR “indebtedness”) OR AB (“gift receipt” OR “receiving gifts” OR “gratitude” OR “gratefulness” OR “thankfulness” OR “indebtedness”)	
2	TI (“gift-giving” OR “mutual aid” OR “donation” OR “donations” OR “volunteerism” OR “volunteering” OR “prosocial” OR “prosociality” OR “generosity” OR “altruism” OR “warm glow”) OR AB (“gift-giving” OR “mutual aid” OR “donation” OR “donations” OR “volunteerism” OR “volunteering” OR “prosocial” OR “prosociality” OR “generosity” OR “altruism” OR “warm glow”)	
3	#1 AND #2	
4	TI (“pay it forward” OR pay-it-forward or “paying it forward” OR paying-it-forward OR “upstream reciprocity” OR “peer donation” OR “peer donations” OR “peer distribution” OR “peer distributions” OR “extended distribution” OR “extended distributions” OR “secondary distribution” OR “secondary distributions” OR “secondary exchange” OR “secondary exchanges” OR “kidney paired donation” OR “kidney paired donations” OR “paired kidney donation” OR “paired kidney donations” OR “paired kidney exchang*” OR “kidney paired exchang*”) OR AB (“pay it forward” OR pay-it-forward or “paying it forward” OR paying-it-forward OR “upstream reciprocity” OR “peer donation” OR “peer donations” OR “peer distribution” OR “peer distributions” OR “extended distribution” OR “extended distributions” OR “secondary distribution” OR “secondary distributions” OR “secondary exchange” OR “secondary exchanges” OR “kidney paired donation” OR “kidney paired donations” OR “paired kidney donation” OR “paired kidney donations” OR “paired kidney exchang*” OR “kidney paired exchang*”)	

© 2025 Ho D et al. *JAMA Network Open*.

5	#3 OR #4	592
6	(((TI ("semi-structured" OR semistructured OR unstructured OR informal OR "in-depth" OR indepth OR "face-to-face" OR structured OR guide OR guides) OR AB ("semi-structured" OR semistructured OR unstructured OR informal OR "in-depth" OR indepth OR "face-to-face" OR structured OR guide OR guides)) AND (TI (interview* OR discussion* OR questionnaire*) OR AB (interview* OR discussion* OR questionnaire*))) OR TI ("focus group" OR "focus groups" OR qualitative OR "mixed methods" OR ethnograph* OR "key informant" OR "participant observation" OR "participant observations" OR anthropology OR anthropological OR narrative OR "grounded theory") OR AB ("focus group" OR "focus groups" OR qualitative OR "mixed methods" OR ethnograph* OR "key informant" OR "participant observation" OR "participant observations" OR anthropology OR anthropological OR narrative OR "grounded theory") OR DE "Interviews" OR DE "Focus Group Interview" OR DE "Intake Interview" OR DE "Interview Schedules" OR DE "Psychodiagnostic Interview" OR DE "Semi-Structured Interview" OR DE "Narratives" OR DE "Qualitative Methods" OR DE "Focus Group" OR DE "Grounded Theory" OR DE "Interpretative Phenomenological Analysis" OR DE "Narrative Analysis" OR DE "Semi-Structured Interview" OR DE "Thematic Analysis" OR DE "Storytelling" OR DE "Digital Storytelling")	
7	#5 AND #6	112
8	#7 AND English	111

Database: Scopus (Elsevier)

Set #		Results
1	TITLE-ABS ("gift receipt" OR "receiving gifts" OR "gratitude" OR "gratefulness" OR "thankfulness" OR "indebtedness")	
2	TITLE-ABS ("gift-giving" OR "mutual aid" OR "donation" OR "donations" OR "volunteerism" OR "volunteering" OR "prosocial" OR "prosociality" OR "generosity" OR "altruism" OR "warm glow")	
3	#1 AND #2	
4	TITLE-ABS ("pay it forward" OR pay-it-forward or "paying it forward" OR paying-it-forward OR "upstream reciprocity" OR "peer donation" OR "peer donations" OR "peer distribution" OR "peer distributions" OR "extended distribution" OR "extended distributions" OR "secondary distribution" OR "secondary distributions" OR "secondary exchange" OR "secondary exchanges" OR "kidney paired donation" OR "kidney paired donations" OR "paired kidney donation" OR "paired kidney donations" OR "paired kidney exchang*" OR "kidney paired exchang*")	
5	#3 OR #4	2587
6	TITLE-ABS ((("semi-structured" OR semistructured OR unstructured OR informal OR "in-depth" OR indepth OR "face-to-face" OR structured OR guide OR guides) AND (interview* OR discussion* OR questionnaire*)) OR "focus group" OR "focus groups" OR qualitative OR "mixed methods" OR ethnograph* OR "key informant" OR "participant observation" OR "participant observations" OR anthropology OR anthropological OR narrative OR "grounded theory")	
7	#5 AND #6	206

© 2025 Ho D et al. *JAMA Network Open*.

8	#7 AND (LIMIT-TO (LANGUAGE , "English"))	200

Database: Sociological Abstracts (ProQuest)

Set #		Results
1	title(“gift receipt” OR “receiving gifts” OR “gratitude” OR “gratefulness” OR “thankfulness” OR “indebtedness”) OR abstract(“gift receipt” OR “receiving gifts” OR “gratitude” OR “gratefulness” OR “thankfulness” OR “indebtedness”)	
2	title(“gift-giving” OR “mutual aid” OR “donation” OR “donations” OR “volunteerism” OR “volunteering” OR “prosocial” OR “prosociality” OR “generosity” OR “altruism” OR “warm glow”) OR abstract(“gift-giving” OR “mutual aid” OR “donation” OR “donations” OR “volunteerism” OR “volunteering” OR “prosocial” OR “prosociality” OR “generosity” OR “altruism” OR “warm glow”)	
3	#1 AND #2	
4	title(“pay it forward” OR pay-it-forward or “paying it forward” OR paying-it-forward OR “upstream reciprocity” OR “peer donation” OR “peer donations” OR “peer distribution” OR “peer distributions” OR “extended distribution” OR “extended distributions” OR “secondary distribution” OR “secondary distributions” OR “secondary exchange” OR “secondary exchanges” OR “kidney paired donation” OR “kidney paired donations” OR “paired kidney donation” OR “paired kidney donations” OR “paired kidney exchang*” OR “kidney paired exchang*”) OR abstract(“pay it forward” OR pay-it-forward or “paying it forward” OR paying-it-forward OR “upstream reciprocity” OR “peer donation” OR “peer donations” OR “peer distribution” OR “peer distributions” OR “extended distribution” OR “extended distributions” OR “secondary distribution” OR “secondary distributions” OR “secondary exchange” OR “secondary exchanges” OR “kidney paired donation” OR “kidney paired donations” OR “paired kidney donation” OR “paired kidney donations” OR “paired kidney exchang*” OR “kidney paired exchang*”)	
5	#3 OR #4	145
6	(((title(“semi-structured” OR semistructured OR unstructured OR informal OR “in-depth” OR indepth OR “face-to-face” OR structured OR guide OR guides) OR abstract(“semi-structured” OR semistructured OR unstructured OR informal OR “in-depth” OR indepth OR “face-to-face” OR structured OR guide OR guides)) AND (title (interview* OR discussion* OR questionnaire*) OR abstract(interview* OR discussion* OR questionnaire*))) OR title(“focus group” OR “focus groups” OR qualitative OR “mixed methods” OR ethnograph* OR “key informant” OR “participant observation” OR “participant observations” OR anthropology OR anthropological OR narrative OR “grounded theory”) OR abstract(“focus group” OR “focus groups” OR qualitative OR “mixed methods” OR ethnograph* OR “key informant” OR “participant observation” OR “participant observations” OR anthropology OR anthropological OR narrative OR “grounded theory”) OR MAINSUBJECT.EXACT.EXPLODE("Qualitative research") OR MAINSUBJECT.EXACT("Interviews") OR MAINSUBJECT.EXACT.EXPLODE("Narratives"))	
7	#5 AND #6	30
8	#7 AND English	27

Database: Social Services Abstracts (ProQuest)

Set #		Results

1	title("gift receipt" OR "receiving gifts" OR "gratitude" OR "gratefulness" OR "thankfulness" OR "indebtedness") OR abstract("gift receipt" OR "receiving gifts" OR "gratitude" OR "gratefulness" OR "thankfulness" OR "indebtedness")	
2	title("gift-giving" OR "mutual aid" OR "donation" OR "donations" OR "volunteerism" OR "volunteering" OR "prosocial" OR "prosociality" OR "generosity" OR "altruism" OR "warm glow") OR abstract("gift-giving" OR "mutual aid" OR "donation" OR "donations" OR "volunteerism" OR "volunteering" OR "prosocial" OR "prosociality" OR "generosity" OR "altruism" OR "warm glow")	
3	#1 AND #2	
4	title("pay it forward" OR pay-it-forward or "paying it forward" OR paying-it-forward OR "upstream reciprocity" OR "peer donation" OR "peer donations" OR "peer distribution" OR "peer distributions" OR "extended distribution" OR "extended distributions" OR "secondary distribution" OR "secondary distributions" OR "secondary exchange" OR "secondary exchanges" OR "kidney paired donation" OR "kidney paired donations" OR "paired kidney donation" OR "paired kidney donations" OR "paired kidney exchang*" OR "kidney paired exchang*") OR abstract("pay it forward" OR pay-it-forward or "paying it forward" OR paying-it-forward OR "upstream reciprocity" OR "peer donation" OR "peer donations" OR "peer distribution" OR "peer distributions" OR "extended distribution" OR "extended distributions" OR "secondary distribution" OR "secondary distributions" OR "secondary exchange" OR "secondary exchanges" OR "kidney paired donation" OR "kidney paired donations" OR "paired kidney donation" OR "paired kidney donations" OR "paired kidney exchang*" OR "kidney paired exchang*")	
5	#3 OR #4	46
6	(((title("semi-structured" OR semistructured OR unstructured OR informal OR "in-depth" OR indepth OR "face-to-face" OR structured OR guide OR guides) OR abstract("semi-structured" OR semistructured OR unstructured OR informal OR "in-depth" OR indepth OR "face-to-face" OR structured OR guide OR guides)) AND (title (interview* OR discussion* OR questionnaire*) OR abstract(interview* OR discussion* OR questionnaire*))) OR title("focus group" OR "focus groups" OR qualitative OR "mixed methods" OR ethnograph* OR "key informant" OR "participant observation" OR "participant observations" OR anthropology OR anthropological OR narrative OR "grounded theory") OR abstract("focus group" OR "focus groups" OR qualitative OR "mixed methods" OR ethnograph* OR "key informant" OR "participant observation" OR "participant observations" OR anthropology OR anthropological OR narrative OR "grounded theory") OR MAINSUBJECT.EXACT.EXPLODE("Qualitative research") OR MAINSUBJECT.EXACT("Interviews") OR MAINSUBJECT.EXACT.EXPLODE("Narratives"))	
7	#5 AND #6	12
8	#7 AND English	12

eTable 1. Study demographics

Study	Study country/countries	Country income level	Type of intervention	Key populations	Study design	Qualitative analysis	Sampling method	Total number of participants interviewed	Breakdown of participants by key population	Mean or median age	N and % female
Agot 2020	Kenya	Lower-middle income	Secondary distribution of HIV self-test kits	Heterosexual couples; Serodiscordant couples	In-depth interview	None mentioned	Criteria sampling	11	All serodiscordant couples	26.36	11, 100%
Balán 2020	United States	High-income	Secondary distribution of HIV self-test kits	Men who have sex with men	In-depth interview	Thematic analysis	Criteria sampling	10	All MSM, mostly racial/ethnic minorities, n/% not specified	36.6, but of 14 participants, not just 10 in interview	0, 0%
Boisvert Moreau 2022	Benin	Lower-middle income	Secondary distribution of HIV self-test kits	Female sex workers	In-depth interview	Thematic analysis; Content analysis	Purposive sampling	29	55.2% had a boyfriend, 27.6% had no education, mostly from Benin (37.9%), average of 3.8 years in sex work, 86.2% biological mothers, 17.2% HIV positive	34.3 (mean)	29, 100%
Brothers 2016	United States	High-income	Secondary distribution of	People who inject drugs /	Semi-structured, in-	Discursive analysis	Criteria sampling	30	10 Black, 19 White, 1	43.5	9 female (30%); 2

© 2025 Ho D et al. *JAMA Network Open*.

			sterile needles	people who use drugs	depth interview				Hispanic; 5 homeless; 10 less than high school education; 16 heroin as drug of choice; 18.5 years injecting; 6 years providing syringes; 501 syringes distributed per week; mean of 23.6 clients		transgender (6.6%)
Bryant 2009	Australia	High-income	Secondary distribution of sterile needles	People who inject drugs / people who use drugs	In-depth interview; Other: Surveys	Other	Convenience sampling	15	12 men, 3 women	Between 26 and 46	3, 20%
Bryant 2019	Australia	High-income	Secondary distribution of sterile needles	People who inject drugs / people who use drugs	In-depth interview	Thematic analysis	Purposive sampling	22	10 people who inject drugs, 6 NSP staff (some peers with history of injection drug use), 6 police	Range from 21 to 63	1, 10% of PWID female; 2, 33% of staff female
Bwalya 2020	Zambia	Lower-midd	Secondary distribu	Heterosexual couples	In-depth interview; Focus	Thematic	Purposive	40 in-depth intervie	Refer to Table 1		17, 51.5% for

© 2025 Ho D et al. *JAMA Network Open*.

		le income	tion of HIV self-test kits		group; Participant observation	analysis	sampling	ws, 91 in focus group discussions, 22 observations			IDIs, 49, 53.8% for FGDs
Byrne 2024	China	Upper-middle income	Pay-it-forward of STI tests	Men who have sex with men	Semi-structured interview	Thematic analysis	Purposive sampling	24	67% had bachelor's degree, 92% not married, 13% completely out	24 (mean)	0, 0%
Dechman 2015	Canada	High-income	Secondary distribution of sterile needles	People who inject drugs / people who use drugs	Focus group	Thematic analysis	Purposive sampling	17			
Grund 1992	Netherlands	High-income	Secondary distribution of sterile needles	People who inject drugs / people who use drugs	Participant observation; Other: Ethnography	Ethnography	None mentioned	N/A			
Holmes 2020	South Africa	Upper-middle income	Secondary distribution of HIV self-test kits	Heterosexual couples; Adolescent girls and young women; Children and adolescents (<19)	Semistructured, in-depth interview	Grounded theory	Purposive sampling	91	Of 80 with quantitative information, 49 AGYW, 31 male partners, with most in steady relationship and not living together	21.1 for AGYW, 26.4 for male partners	Of 80 with quantitative information, 49, 61.3%
Ky-Zerbo 2022	Côte d'Ivoire, Mali, Senegal	Lower-middle income;	Secondary distribution of HIV self-	Female sex workers	Focus group	Thematic analysis	Convenience sampling	87		27	87, 100%

© 2025 Ho D et al. *JAMA Network Open*.

		Low-income	test kits								
Ky-Zerbo 2022	Côte d'Ivoire, Mali, Senegal	Lower-middle income; Low-income	Secondary distribution of HIV self-test kits	Men who have sex with men; Female sex workers; People who inject drugs / people who use drugs	Semi-structured interview	Thematic analysis	Criteria sampling	89	21 MSM, 24 FSW, 20 PWUD; 24 secondary recipients	25 for MSM, 27 for FSW, 44 for PWUD, 25 for secondary recipients through MSM-based channel, 32 for secondary recipients through FSW-based channel	24 FSW, 3 PWUD, 3 secondary recipients
Li 2020	China	Upper-middle income	Pay-it-forward of STI tests	Men who have sex with men	Semi-structured interview	Thematic analysis	Convenience sampling	30		28	0, 0%
Maman 2017	Kenya	Lower-middle income	Secondary distribution of HIV self-test kits	Female sex workers	In-depth interview	None mentioned	Purposive sampling	18		28.4	18, 100%
Matovu 2018	Uganda	Low-income	Secondary distribution of HIV self-test kits	Heterosexual couples; Pregnant women	In-depth interview	Thematic analysis	Purposive sampling	32	17 pregnant women, 15 male partners		17, 53.1%
Murphy 2004	United States	High-	Secondary distribu	People who inject	In-depth interview;	None mentioned	Other: Maximum	244		38	94, 38.6%

© 2025 Ho D et al. *JAMA Network Open*.

		income	tion of sterile needles	drugs / people who use drugs	Participant observation		variation sampling				
Napierala 2019	Malawi, Zimbabwe	Lower-middle income; Low-income	Secondary distribution of HIV self-test kits	Female sex workers	Semi-structured interview; Focus group; Participant observation; Other: Daily event reporting diary	Thematic analysis	Purposive sampling	At least 267 in FGDs, IDIs			267, 100%
Newland 2016	Australia	High-income	Secondary distribution of sterile needles	People who inject drugs / people who use drugs	Semi-structured, in-depth interviews; Other: Social network mapping	Thematic analysis	Snowball sampling	32		31	12, 37.5%
Rael 2020	United States	High-income	Secondary distribution of HIV self-test kits	Transgender individuals	In-depth interview	None mentioned	Convenience sampling	10		28.4	10, 100%
Ruderman 2022	Kenya	Lower-middle income	Secondary distribution of HIV self-test kits	Heterosexual couples; Female sex workers	In-depth interview	Thematic analysis	Purposive sampling	32	15 from sex work hot spots, 17 from beach communities	26.6	32, 100%
Sha 2023	China	Upper-middle income	Secondary distribution of HIV self-test kits	Men who have sex with men	Semi-structured, in-depth interview	Thematic analysis	Purposive sampling	22			0, 0%
Snead 2003	United States	High-income	Secondary distribution of	People who inject drugs /	Semi-structured interview	Grounded theory	Snowball sampling	47	26 primary distributors	52 for providers, 47 for	14, 30%

© 2025 Ho D et al. *JAMA Network Open*.

			sterile needles	people who use drugs					(givers), 21 recipients	recipients	
Strike 2005	Canada	High-income	Secondary distribution of sterile needles	People who inject drugs / people who use drugs	Semi-structured, in-depth interview	Other	Purposive sampling	120			
Voytek 2003	United States	High-income	Secondary distribution of sterile needles	People who inject drugs / people who use drugs	Semi-structured interview	None mentioned	None mentioned	30	20 primary distributors ("givers"), 10 recipients		11, 37%
Wang 2024	China	Upper-middle income	Secondary distribution of HIV self-test kits	Men who have sex with men	Focus group; Other: Photovoice	Other	Purposive sampling	22	18 gay, 3 bisexual, 1 unsure/did not want to share	28 years	0, 0%
Ware 2023	Uganda	Low-income	Secondary distribution of HIV self-test kits	Heterosexual couples; Serodiscordant couples; Pregnant women	In-depth interview	Other	Purposive sampling	59	29 women, 30 male partners	27 for women, 32 for men	29, 49.1%

© 2025 Ho D et al. *JAMA Network Open*.

eTable 2. Representative quotes for themes

Category	Theme	Representative quotes
**Motivations**	Selfless concern for others was a major motivation for givers, often seen as a moral prerogative.	"My partner loves me so much that she brought me an HIV self-test kit so that I can know where I stand. – MP, accepted test and shared HIV results."[33]<br><br>"[The client] told me that I care about his health apart from the money I'm looking for, that means I consider him, that I want him to be healthy… It was the way he liked it that made me do [the self-test] on others."[31]<br><br>"There should be no reason [to share or reuse needles]. I look at it this way, I do it 24 hours a day because there is no reason—as long as I am in this city—that anybody should have to use a dirty needle."[44]<br><br>"For example, Chris (male, 21 years) from Newcastle explained ''I loved it because at least then I knew that everyone had [sterile equipment]."[50]<br><br>"Because of the paying forward of love, I am willing to give a little of my love and make some contributions to the next person…"[54]
	Givers felt a profound sense of empowerment from taking on a prosocial identity as a public health agent.	"[Syringe exchange program staff] argued that the authorisation had important symbolic meaning by legitimising the care that drug users show for themselves and their peers, demonstrating that 'we are not selfish addicts, self-centred and unkind people and that we do give a f*** about each other and are willing to make sure that we are all looked after within our group.' (Staff 5)."[46]<br><br>"Several FSWs said they were proud to be role models for others, by sharing HIV self-tests, teaching how to use the device and directly assisting secondary beneficiaries."[31]<br><br>"Women demonstrated increased confidence in the use of HIVST with partners over time. This confidence resulted in increased agency to negotiate condom use with partners deemed to be high-risk and to refuse sex or end relationships with those partners who refused condom use."[40]
	Though not common, distribution or participation in donation-based interventions could occur with transactional intentions.	"I'll sell like, when people cant get 'em, they'll pay like a dollar for 1 point. So, I can sell a pack for 5 to 10 dollars depending on how desperate someone is."[44]<br><br>"Only one individual explicitly mentioned profiting from the generosity of others: "Originally the price is 150, I donated 10, just took advantage.""[55]
**Facilitators**	Social norms of sharing normalized givers distributing health services.	"They see themselves as helping the members of their community, and many also provide money, food, and other forms of support, in addition to syringes, to their clients."[44]

		"Secondary distribution to peers seems "natural" because primary users are convinced that all of them are exposed to the same degree of risk."[35]
	Social proximity between giver and recipient enabled tailored strategies to introduce the health service, strengthen relationships, and engage in reciprocal giving.	"Providers most often reported having close, long-term social relationships with recipients—many were friends or roommates, and several were family members or lovers. "We went to school together and prison together, and we're pretty close.... Most [of my recipients] know each other." "It's not just anybody... just my friends...my sister and brother.""[51]<br><br>"Among participants who wanted their romantic sexual partners to self-test, since most participants and their partners took regular HIV test or had prior experience talking about sexual health, they had no difficulty persuading their partner to take the self-testing kit."[41]<br><br>"We were a happy family, yes, but there is some kind of situation where being together, talking together as husband and wife was difficult but from that time... we usually sit down, talk, discuss and see the way forward on how to manage our family, to take care of our family."[29]<br><br>"Because he said [to give it to] my partners and their partners, because as it is, in the community, I may know someone who also knows someone, and so the distribution is done. So, I take it and I give it to my partners who will give it to their partners, and so on."[35]
**Barriers**	Secondary syringe exchange could put too much risk and responsibility on givers, leading to legal harms and informal doctoring burdens.	"In their efforts to keep users alive, their practices of informal doctoring extend far beyond their officially sanctioned roles of disseminating sterile syringes and safe injection information. They reluctantly inject those who are unable to safely inject themselves, lance and care for abscesses, save and share antibiotics and other drugs, act as councillors, encourage withdrawal if possible, and offer temporary housing."[47]<br><br>"Some providers refused to inject people because they did not want to encourage drug use or would only inject those who they had known to be IDUs, and several reported trying to discourage others from using drugs."[51]<br><br>"But the reason we told them [needle recipients] to go as far [away] as they can is because if they do get busted, and they do get asked where did you get your stuff from, and they can point out our place, you know, we are homeless instantly. (Client 2)."[46]
	Social harms from HIVST distribution were present but generally rare.	"Some women experienced physical violence, mostly perpetrated by an established partner, linked to disclosure of results or requesting that a partner also test and typically in the context of pre-existing relationship violence."[38]<br><br>"He took it negatively... he changed and told me to keep quiet, and he pushed me immediately on the bed, and told me those words that, he has to sleep with me without a condom, since there is no where I can take him."[36]
