# Supplementary material for: Motivations, Facilitators, and Barriers of Donation-Based Interventions in HIV and Sexually Transmitted Infection Research: A Systematic Review
Source: JAMA Netw Open. 2025 Oct 14;8(10):e2537382. doi: 10.1001/jamanetworkopen.2025.37382 (PMC12522005; doi:10.1001/jamanetworkopen.2025.37382)
Supplement: Supplement 2. — Data Sharing Statement [file jamanetwopen-e2537382-s002.pdf]

Data Sharing Statement

Data
Data available: No

Additional Information
Explanation for why data not available: The data in this systematic review and qualitative evidence synthesis is secondary data; we do not have any patient data to share.
